# Clinical observation of sacubitril valsartan sodium in the treatment of resistant hypertension: A randomized clinical trial

**DOI:** 10.3389/fcvm.2022.1099043

**Published:** 2022-12-16

**Authors:** Tian-Jiao Lyu, Ying Liu, Hong Zhang, Ling-Yan Li, Rui-Qing He, Jun-Qing Gao, Zong-Jun Liu

**Affiliations:** Department of Cardiology, Putuo Hospital, Shanghai University of Traditional Chinese Medicine, Shanghai, China

**Keywords:** sacubitril valsartan sodium, resistant hypertension (RH), blood pressure, myocardial remodeling, clinical observation

## Abstract

**Objective:**

To investigate the effectiveness and safety of sacubitril valsartan sodium in the treatment of resistant hypertension (RH).

**Methods:**

This study is a single-center, prospective, randomized controlled study. According to the inclusion and exclusion criteria, patients with RH who met the criteria were screened, and all patients adjusted their drug treatment (valsartan 80 mg, amlodipine 5 mg, and hydrochlorothiazide 12.5 mg). After 4 weeks of drug elution, the random envelope method was used for random grouping. The treatment group took sacubitril valsartan sodium 200 mg, amlodipine 5 mg, hydrochlorothiazide 12.5 mg, and the control group took valsartan 80 mg, amlodipine 5 mg, and hydrochlorothiazide 12.5 mg for 8 weeks. The 24 h ambulatory blood pressure (BP) and the echocardiography index using the office sphygmomanometer were observed in the patients.

**Results:**

A total of 100 patients with RH were included in the two groups, with 50 cases in each group. There were no significant differences in sex, age, or comorbid diseases between the two groups. During the 8-week follow-up, the office BP of the research group were significantly decreased (24.78/17.86 mmHg) compared with those of the control group. In the research group the 24 h average BP, daytime average BP, and nighttime average BP were 144.84/79.82, 147.10/82.06, and 138.67/76.31 mmHg at baseline, and reduced to 128.96/73.32, 131.50/74.94, and 122.11/69.27 mmHg at week 8, which were significantly decreased (*P* < 0.05 or *P* < 0.01), and the left ventricular ejection fraction was significantly increased (*P* < 0.05), compared with the control group.

**Conclusion:**

Sacubitril valsartan sodium can effectively reduce BP and improve cardiac function in RH.

## 1 Introduction

Resistant hypertension (RH) means that based on improving lifestyle, taking three or more antihypertensive drugs (including a diuretic) blood pressure (BP) has not reached the target (> 140/90 mmHg) (1–4). It is caused by a variety of pathogenic factors separately or together. The prevalence of RH is 5–15% and poor control of BP induces long-term target organ damage, including heart, brain, and kidney damage, leading to an increased incidence of major adverse cardiac events and affecting the quality of life and clinical prognosis (5, 6). Therefore, the treatment of RH has always been a challenge in the field of high BP. Sacubitril valsartan sodium, which is combined with the neprilysin inhibitor sacubitril and the angiotensin receptor blocker valsartan, has become the first-line drug for the treatment of ejection fraction decrease in heart failure (7). It was added to the international hypertension guidelines for the first time in 2020. A study confirmed that sacubitril valsartan has fast effective antihypertensive efficacy for early or incipient hypertension. Sacubitril valsartan sodium, through two pathways, resists the excessive activation of neuroendocrine cells and inhibits the release of renin and aldosterone, which may be the key target for the treatment of RH (8, 9). Therefore, we conducted a prospective randomized controlled study on the treatment of RH with sacubitril valsartan (Registration number: ChiCTR1900027727).

## 2 Materials and methods

This research project is a prospective, randomized controlled clinical trial. A total of 100 patients with RH were enrolled. The follow-up time was 8 weeks.

### 2.1 Inclusion criteria

(1) Patients with RH aged 18–80.
(2) Patients whose BP had not reached the standard after drug treatment [24-h average systolic blood pressure (SBP) ≥ 140 mmHg or average diastolic blood pressure (DBP) ≥ 90 mmHg, and office SBP ≥ 140 mmHg or office DBP ≥ 90 mmHg].
(3) Voluntarily participate in this research and signature of the informed consent form.

### 2.2 Exclusion criteria

(1) Secondary hypertension.
(2) Severe renal impairment (eGFR < 30 ml/min/1.73 m^2^).
(3) Severe liver damage (Child-Pugh grade C).
(4) Patients with type 2 diabetes who consume alcohol.
(5) Patients with a known history of angioedema related to angiotensin-converting enzyme inhibitor (ACEI) or angiotensin II receptor blocker (ARB) treatment.
(6) Patients with hereditary or idiopathic angioedema.
(7) Patients who were pregnant or planning to become pregnant during the study.

### 2.3 Study design

**FIGURE 1:**
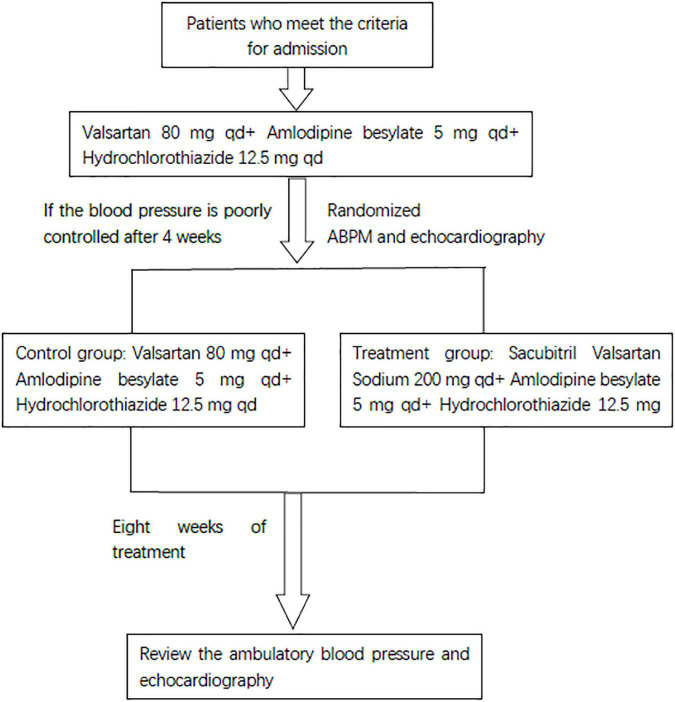
Study design.

Patients who met the RH diagnosis standard started the following treatment for at least 4 weeks: valsartan 80 mg, once a day, 80 mg each time, amlodipine besylate, once a day, 5 mg each time, hydrochlorothiazide 12.5 mg, 12.5 mg once a day, orally. Patients whose BP was still poorly controlled (24-h average SBP ≥ 140 mmHg or average DBP ≥ 90 mmHg, and office SBP ≥ 140 mmHg or office DBP ≥ 90 mmHg) after at least 4 weeks of treatment completed a check-up study. The randomized envelope method was used in this study. Then, the patients were randomly divided into the research group and the control group. The control group continued the above drug treatment, but the research group stopped taking valsartan and started sacubitril valsartan sodium (the sacubitril 98 mg/valsartan 102 mg) instead, 200 mg once a day and continued to take the other two drugs. The two groups of patients continued the regimen for 8 weeks. The study design is shown in Figure 1. The primary endpoint was a composite of office BP, ambulatory BP, and echocardiography.

### 2.4 Office BP and ambulatory BP measurements

Office BP was measured by an automated BP monitor (Omron HEM-7124, Dalian, China) with appropriately size cuffs in conformity to the 2018 Chinese guidelines for prevention and treatment of hypertension (10). BP measurements should be taken 1–2 min apart and averaged for records. An additional measurement is required if the first two readings differ by > 5 mmHg, and the mean value of the three readings should be recorded.

The ambulatory BP was measured by an ambulatory BP monitor (TM-2430, A&D, Japan). The ambulatory BP monitoring time was from 6:00 to 21:59 for daytime and from 22:00 to 5:59 for nighttime. Measurements were performed per 30 min during the day and per hour during the night. Patients were told to avoid strenuous exercise during the monitoring. Ambulatory BP monitoring was performed with all patients enrolled at the baseline and week 8.

### 2.5 Statistical methods

All data were analyzed with SPSS 22.0 software, and the measurement data conforming to a normal distribution were expressed as the mean ± standard deviation ($\bar{X}$ ± S). The independent samples *t*-test was used to compare the two groups. The paired-test was used to compare the two groups before and after treatment. The enumeration data adopted the *x*^2^ test.

## 3 Results

### 3.1 Baseline demographics and characteristics

This study included 100 patients with RH. They were randomly divided into the research group and control group, with 50 cases in each group. All patients completed the treatment and follow-up. There were no sex differences between the two groups of patients. The average age of the patients in the research group was 70.08 ± 9.29, and that of the control group was 70.56 ± 7.12 years. There were no significant differences between them. Both groups of patients had coronary heart disease, diabetes, heart failure, atrial fibrillation, or stroke, but there was no obvious difference between them. The blood biochemical indicators shown in Table 1 showed no obvious differences.

**TABLE 1:** Baseline demographic characteristics.

	Research group (*n* = 50)	Control group (*n* = 50)	*P*
**Gender (*n*)**			0.841
Male (%)	24 (48%)	25 (50%)	
Female (%)	26 (52%)	25 (50%)	
Age (year)	70.08 ± 9.29	70.56 ± 7.12	0.773
**Merger disease (*n*)**			
Coronary heart disease	12 (24%)	13 (26%)	0.817
Atrial fibrillation	6 (12%)	5 (10%)	0.749
Stroke	7 (14%)	6 (12%)	0.766
Diabetes	12 (24%)	11 (22%)	0.812
Heart failure	11 (22%)	12 (24%)	0.812
Hemoglobin (g/L)	129.84 ± 15.416	136.80 ± 17.50	0.125
Glutamic pyruvic transaminase (U/L)	22.56 ± 13.09	21.12 ± 10.11	0.647
Glutamic oxaloacetic transaminase (U/L)	24.22 ± 10.41	24.32 ± 8.01	0.969
Creatinine (μmol/L)	82.82 ± 41.02	70.86 ± 19.68	0.170
Uric acid (μmol/L)	349.00 ± 90.59	326.68 ± 96.19	0.375
Total cholesterol (mmol/L)	4.63 ± 1.28	4.78 ± 0.88	0.598
Low-density lipoprotein (mmol/L)	3.00 ± 1.02	3.13 ± 0.70	0.600
High-density lipoprotein (mmol/L)	1.15 ± 0.24	1.12 ± 0.31	0.716
Triglycerides (mmol/L)	1.62 ± 0.96	1.71 ± 0.88	0.710
Fasting plasma glucose (mmol/L)	6.91 ± 3.55	6.01 ± 1.97	0.253
Serum potassium (mmol/L)	4.17 ± 0.50	3.93 ± 0.45	0.068
Serum sodium (mmol/L)	141.03 ± 4.93	141.77 ± 4.14	0.552
Serum chloride (mmol/L)	104.85 ± 4.36	104.85 ± 4.36	0.833
			

### 3.2 Office BP and ambulatory BP measurements

**FIGURE 2:**
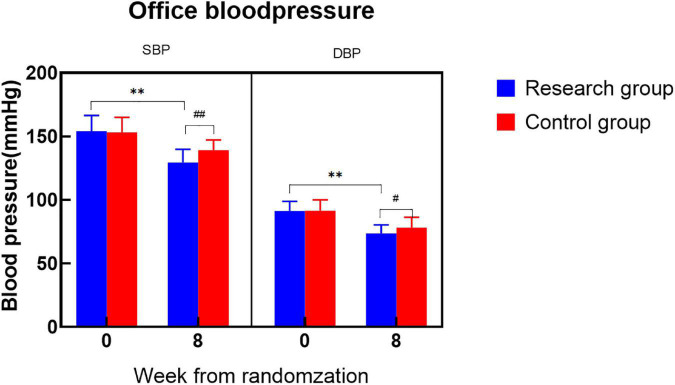
Office blood pressure.

During the 8-week follow-up, the systolic and DBP of the research group were significantly decreased (24.78/17.86 mmHg) compared with those of the control group (Figure 2).

There was no significant difference in the baseline office BP between the two groups of patients, and they were comparable. At the 8-week follow-up, the office BP of the two groups was significantly lower than the baseline office BP (^**^P < 0.001). The office BP in the control group decreased by 14.1/13.3 mmHg, while the office BP in the research group decreased by 24.78/17.86 mmHg, which was more significant than that in the control group (^#^*P* < 0.05, ^##^*P* < 0.001).

**FIGURE 3:**
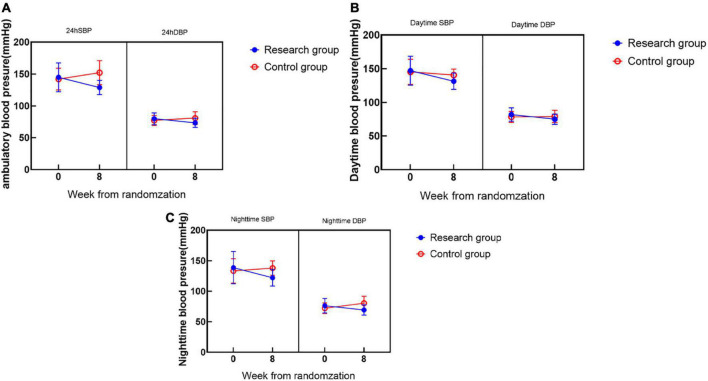
Ambulatory blood pressure.

During the 8-week follow-up, the 24-h average BP, daytime average BP, and nighttime average BP in the two groups were significantly lower than those before treatment, with significant differences (Figure 3).

There was no significant difference in the baseline ambulatory BP between the two groups of patients, and the results were comparable. At the 8-week follow-up, the 24-h mean BP in the research group (SBP 128.96 ± 11.11 mmHg, DBP 73.32 ± 7.16 mmHg) was significantly lower than that at 0 weeks (SBP 144.84 ± 22.53 mmHg, DBP 79.82 ± 9.38 mmHg) (^**^P < 0.001) and decreased more significantly than that in the control group (^##^*P* < 0.001) (Figure 3A); the daytime mean BP (SBP 131.50 ± 12.05 mmHg, DBP 74.94 ± 7.73 mmHg) in the research group was significantly higher than that at 0 weeks (SBP 147.10 ± 21.43 mmHg, DBP 82.02 ± 10.06 mmHg) and decreased significantly (^**^P < 0.001) and was more significant than that in the control group (^#^*P* < 0.05) (Figure 3B); the mean nighttime BP in the research group (SBP 122.11 ± 13.61 mmHg, DBP 69.27 ± 8.40 mmHg) was significantly lower than that at the 0 week (SBP 138.67 ± 26.40 mmHg, DBP 76.31 ± 11.84 mmHg) (^**^P < 0.001), and was more significant than the control group (^#^*P* < 0.05) (Figure 3C).

### 3.3 Echocardiography

There was no significant difference in the baseline levels of the two groups of patients at 0 weeks. At the 8-week follow-up, the left ventricular ejection fraction (55.08 ± 8.17%) in the research group was significantly higher than that at 0 weeks (48.36 ± 6.89%) (^**^P < 0.001) and better than that in the control group (^#^*P* < 0.05), with a significant difference; the left atrial diameter in the research group (37.04 ± 5.91 mm) was significantly lower than that at 0 weeks (38.94 ± 4.54 mm) (*P < 0.05), E/e′ (10.01 ± 4.14) was significantly lower than that at 0 weeks (11.80 ± 5.09) (*P < 0.05), but there was no significant difference compared with that in the control group; the thickness of the interventricular septum in the research group (9.00 ± 2.04 mm) was significantly smaller than that in the control group (10.22 ± 1.21 mm) (^#^*P* < 0.05), but there was no significant difference between the two groups. There were no significant differences in the left ventricular end-diastolic diameter, left ventricular systolic, left ventricular posterior wall thickness, or pulmonary artery pressure after treatment (Table 2).

**TABLE 2:** Echocardiography.

	Research group	Control group	*P*
**LVEF (%)**			
0 week	48.36 ± 6.89	49.98 ± 16.21	0.220
8 week	55.08 ± 8.17**^,#^	50.69 ± 9.87	0.018
**LVEDd (mm)**			
0 week	49.37 ± 8.14	49.56 ± 4.56	0.885
8 week	51.44 ± 7.01	50.52 ± 6.39	0.505
**LVESd (mm)**			
0 week	34.73 ± 6.13	35.15 ± 4.97	0.718
8 week	36.24 ± 6.94	35.39 ± 6.95	0.551
**IVST (mm)**			
0 week	8.76 ± 4.23	9.65 ± 3.14	0.243
8 week	9.00 ± 2.04^#^	10.22 ± 1.21	0.001
**LVPW (mm)**			
0 week	8.12 ± 3.90	9.20 ± 3.02	0.129
8 week	9.02 ± 1.39	9.54 ± 1.29	0.060
**LA (mm)**			
0 week	38.94 ± 4.54	39.00 ± 4.56	0.947
8 week	37.04 ± 5.91*	38.78 ± 4.48	0.109
**E/e′**			
0 week	11.80 ± 5.09	11.93 ± 4.27	0.896
8 week	10.01 ± 4.14*	10.56 ± 4.76	0.582
**FS (%)**			
0 week	31.00 ± 9.28	29.21 ± 5.62	0.257
8 week	29.80 ± 5.58	29.26 ± 6.86	0.673
**PAP (mmHg)**			
0 week	34.33 ± 8.90	32.94 ± 7.74	0.508
8 week	32.52 ± 17.35	32.75 ± 9.32	0.946

*P < 0.05, compared with before treatment, **P < 0.001, compared with control group, the ^#^*P* < 0.05, ^##^*P* < 0.001.

### 3.4 Correlation analysis between BP and echocardiographic indices

Ultrasound septal thickness and 24 h mean SBP (*r* = 0.296, *P* = 0.003 < 0.05), daytime mean SBP (*r* = 0.238, *P* = 0.020 < 0.05) and nighttime mean SBP (*r* = 0.316, *P* = 0.003 < 0.05) were significantly positively correlated (Table 3).

**TABLE 3:** Correlation analysis between blood pressure (BP) and echocardiographic indices.

	LVEF (%)	IVST (mm)	LA (mm)	E/e′
**24 h mean ambulatory SBP (mmHg)**				
*R*	−0.116	0.296^▲▲^	0.169	0.068
*p*	0.253	0.003	0.101	0.548
**24 h mean ambulatory DBP (mmHg)**				
*R*	−0.154	0.148	0.014	−0.025
*P*	0.127	0.150	0.889	0.827
**Daytime mean ambulatory SBP (mmHg)**				
*R*	−0.069	0,238^▲^	0.134	0.062
*P*	0.459	0.020	0.195	0.584
**Daytime mean ambulatory DBP (mmHg)**				
*R*	−0.155	0.133	0.138	0.048
*P*	0.124	0.197	0.179	0.672
**Nighttime mean ambulatory SBP (mmHg)**				
*R*	−0.106	0.316^▲^	0.099	0.007
*P*	0.124	0.003	0.358	0.953
**Nighttime mean ambulatory DBP (mmHg)**				
*R*	−0.080	0.261^▲^	0.002	0.000
*P*	0.453	0.014	0.987	0.999

^▲▲^At the 0.01 level significantly correlated; ^▲^at the 0.05 level.

## 4 Discussion

Resistant hypertension refers to the condition in which taking three or more antihypertensive drugs (including a diuretic) and improving lifestyle, do not reduce BP to the standard (> 140/90 mmHg). These patients generally have severe target organ damage accompanied by dizziness, headache, tinnitus, irritability, insomnia, and other symptoms. They are very prone to renal insufficiency, myocardial infarction, stroke, heart failure, and other complications, and the prognosis is poor. Because RH is difficult to control with drugs, its treatment has always been a major challenge in the field of hypertension. Studies have shown (11) that the enhanced and persistent activity of the sympathetic nerve and the renin-angiotensin-aldosterone system (RAAS) is one of the important pathogenic mechanisms of RH. The activation of the RAAS and the excessive increase in sympathetic nerve activity can initiate the process of inflammatory factors and oxidative stress and at the same time promote the occurrence and development of arteriosclerosis and atherosclerosis, aggravate abnormalities in vascular structure and function, and make it difficult to control hypertension. At present, the basic drug treatment for RH is RAAS blocker (ARB or ACEI) combined with a calcium ion blocker (CCB) and thiocyanate. Triple therapy with azine diuretics is the mainstay.

Sacubitril valsartan sodium is a dual preparation of enkephalinase and an angiotensin receptor antagonist. Sacubitril valsartan sodium inhibits enkephalinase through LBQ657 (the active metabolite of the prodrug sacubitril) (neutral endopeptidase; NEP) while blocking the angiotensin II type 1 receptor (AT1) by valsartan. Both pathways can counteract neuroendocrine overactivation and inhibit the release of renin and aldosterone, resulting in vasodilation, inhibition of cardiac hypertrophy, reduction of cardiac preload, and improvement of ventricular remodeling. Currently, several guidelines (12, 13) recommend the use of sacubitril and valsartan sodium for the treatment of hypertension.

This study is a clinical study on the treatment of RH with sacubitril valsartan sodium. The randomized controlled method was used to observe the efficacy of sacubitril valsartan sodium in the treatment of RH. The 100 patients enrolled were all patients with RH, and after 4 weeks of standardized triple antihypertensive treatment, their BP remained poorly controlled. They passed the initial screening and entered the follow-up treatment. One hundred patients were randomly divided into a research group and a control group. There was no significant difference in the baseline conditions between the two groups before treatment, so the results of the study were comparable. The results suggest that sacubitril valsartan sodium combined with amlodipine besylate and hydrochlorothiazide significantly reduces office BP, 24-h average BP, daytime average BP, and nighttime average BP in patients with RH indicating that sacubitril valsartan sodium can better control BP in patients with RH. Li et al. (14) studied 66 patients with RH, used sacubitril and valsartan sodium instead of other ACEI/ARB drugs, and continued other antihypertensive drugs taken by the patients at the same time. The results demonstrated that sacubitril valsartan sodium can significantly reduce office BP and ambulatory BP in patients with RH, which is consistent with our findings. However, we adopted a randomized controlled study method with larger sample size, and all the enrolled patients regularly took the same antihypertensive drugs during the initial screening period. In addition, other concomitant antihypertensive drugs were also consistent, which provided a balanced comparison between the groups, effectively reducing the influence of potential unknown factors on the experimental results and making the research results more convincing.

In addition to the difficulty reaching the BP target, patients with RH are also prone to damage to other target organs, and the risk of cardiovascular disease is significantly increased. Therefore, the treatment of RH should take into account both potent antihypertensive and target organ protection. Several guidelines also recommend the use of sacubitril and valsartan sodium for the treatment of patients with hypertension and cardiovascular disease (14, 15). In the 2022 ACC/AHA heart failure management guidelines (16), it was proposed that people with “precursor heart failure” should pay attention to prevention, and optimizing BP control is one of the key points. Improving cardiac remodeling and protecting cardiac function is also an important reason why we chose to use sacubitril and valsartan sodium in combination with other drugs to treat RH. While comparing the BP control of the two groups of patients, we also performed echocardiography. The results of the study showed that sacubitril-valsartan sodium combined with amlodipine besylate, and hydrochlorothiazide could improve left ventricular ejection fraction, reduce E/e′, reduce left atrial diameter, and improve cardiac systolic and diastolic function.

The effect of sacubitril and valsartan sodium in the treatment of RH may be related to its new antihypertensive mechanism, which can exert a stronger antihypertensive effect than traditional antihypertensive drugs. Sacubitril and valsartan sodium are dual preparations of enkephalinase and a vascular angiotensin receptor antagonist that can counteract neuroendocrine overactivation through two pathways and inhibit the release of renin and aldosterone. This could be the key target for the treatment of RH.

This study is a single-center study with an observation period of 8 weeks, which is short, and the conclusions are still limited. The clinical efficacy of sacubitril valsartan sodium in the treatment of RH still needs multicenter and large-scale long-term observation.

## Data availability statement

The original contributions presented in this study are included in the article/supplementary material, further inquiries can be directed to the corresponding authors.

## Ethics statement

The studies involving human participants were reviewed and approved by the Putuo Hospital, Shanghai University of Traditional Chinese Medicine. The patients/participants provided their written informed consent to participate in this study.

## Author contributions

T-JL, YL, and J-QG conceived and designed the study. J-QG and Z-JL bore overall responsibility for the design, ethical conduct, and publication of the study. All authors are involved in the protocol discussion, took responsibility for the study data gathering and verification, edited, drafted, and contributed substantially to the manuscript, and approved this submission.
